# Jejunal perforation due to ingested buffalo bone mimicking acute appendicitis

**DOI:** 10.1186/s13104-016-2127-y

**Published:** 2016-06-24

**Authors:** Ajay Kumar Yadav, Gyanendra Malla, Kunal Bikram Deo, Saroj Giri, Bishnu Murti Bhattarai, Shailesh Adhikary

**Affiliations:** Department of General Practice and Emergency Medicine, B. P. Koirala Institute of Health Sciences, Dharan, Nepal; Department of General Surgery, B.P. Koirala Institute of Health Sciences, Dharan, Nepal

**Keywords:** Local peritonitis, Jejunum perforation, Foreign body, Buffalo bone, Case report

## Abstract

**Background:**

Foreign body ingestion is seen quite frequently in clinical practice, intestinal perforation due to this is rare. The foreign body often mimics another cause of acute abdomen and requires an emergency surgical intervention. The majority of patients do not recall ingesting sharp foreign bodies.

**Case presentation:**

We report an interesting case of a fifty five year-old man who presented with pain in the right iliac fossa with localised tenderness which was clinically diagnosed as acute appendicitis. During the operation, the presence of purulent collection and the inflamed bowel with flakes raised suspicion of bowel perforation. The assessment of the proximal small bowel revealed two small perforations in the jejunum. A hard, bony and sharp object was extracted and the perforations were closed. Post-operative recovery was uneventful. Detailed food history was taken following the recovery of the patient from surgery. It revealed the history ingestion of home prepared buffalo meat. The extracted object was identified as ‘buffalo bone’ by the patient and the care taker of the patient. The jejunum was perforated by the ingested buffalo bone causing local peritonitis in right iliac fossa.

**Conclusion:**

Intestinal perforation by ingested foreign bodies should be suspected in acute abdomen. It requires a high degree of suspicion and awareness on the part of the clinician.

**Electronic supplementary material:**

The online version of this article (doi:10.1186/s13104-016-2127-y) contains supplementary material, which is available to authorized users.

## Background

The majority of ingested foreign bodies (IFB) are excreted from the digestive tract without any complications or morbidity; however, occasionally they may lead to serious clinical problems, such as obstruction, perforation or bleeding [1–3]. Although IFB are quite common problem in children, they are infrequently encountered in adults but are seen in elderly people wearing dentures, alcoholics and/or patients with learning difficulties [4]. IFB, such as chicken bones, fish bones, toothpicks and dentures require surgical intervention in 5 % of cases. Patients are not usually aware of the IFB which is usually detected either during laparotomy or at the time of pathological examination of the surgical specimen [5]. Less than 1 % of IFB, especially large, sharp and/or pointed objects, cause bowel perforation. Perforation usually occurs at the narrowest part of the bowel, either at the ileocecal valve or at the rectosigmoid junction [6]. Sharp fish bone causing perforation in the duodenum has been reported [7]. This case report highlights the importance of detailed history taking for food ingestion in a case presenting with symptoms of acute appendicitis where the pain characteristics are not very typical of appendicitis.

## Case presentation

A fifty five year-old man presented to the emergency room (ER) with a localized pain at the right iliac fossa. There were no histories of nausea, vomiting or any other complaints associated with the pain. History taking revealed that the pain was colicky in nature around the epigastric region when it started 3 days ago. The pain increased gradually by the next day, which eventually localized at the right iliac fossa before the case presented to our ER.

**Fig. 1:**
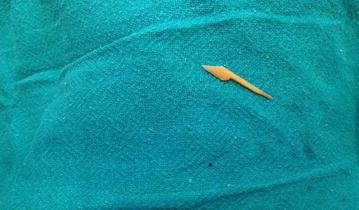
The foreign body: a buffalo bone

**Fig. 2:**
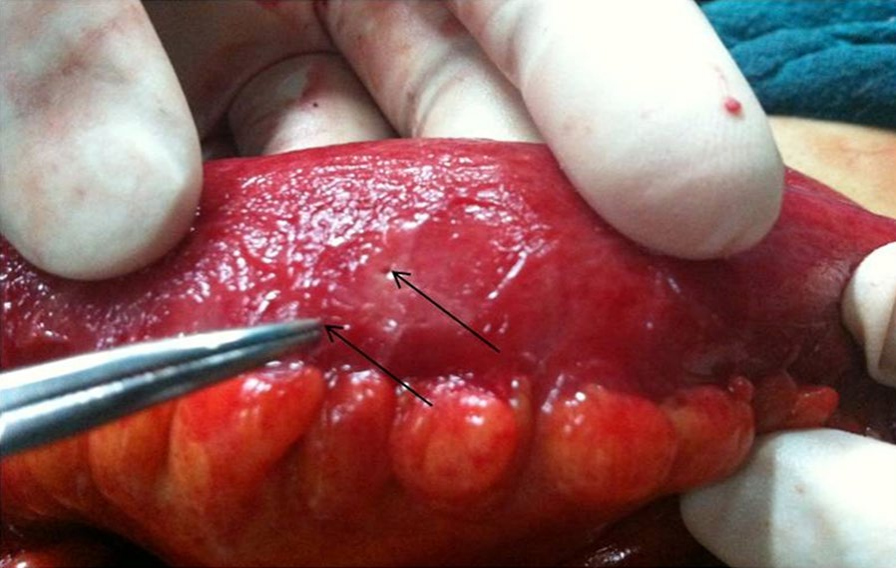
Two tiny jejunal perforations (indicated by the *tip of the arrows*)

The pain at the right iliac fossa was a continuous type which was aggravated by any kind of movement and was relieved by rest. The patient did not give any past history of medical or surgical problems. There were no known drug allergies or use of any medications. He was afebrile. Abdominal examination revealed localised tenderness and with guarding in the right iliac fossa. The baseline work up was normal. The appendix was not visualized on ultrasonogram but revealed minimal fluid collection in the right iliac fossa. A diagnosis of acute appendicitis was done and a plan for emergency appendectomy was made. During operation, Gridiron incision was made to visualize the appendix, which was mildly inflamed. On cut section, the mucosa was found to be inflamed and the presence of a faecolith was noted. Following appendectomy, the presence of inflamed bowel with food flakes and purulent collection raised suspicion of intestinal perforation. We decided to explore the entire bowel. The incision was converted to Rutherford Morrison incision. Bowel examination revealed two small jejunal perforations around 2 mm size in the antimesenteric border, approximately 250 cm proximal to ileocaecal junction. On palpation of the adjacent bowel, a hard, bony and sharp object was felt. This object was removed through the perforation site. The perforations were repaired in two layers with 3/0 polygalactin and 3/0 silk sutures. A pelvic drain was placed, after which the abdomen was closed in layers. Postoperative recovery was uneventful. Histopathology report of the appendix revealed eosinophils, which indicated receding appendicitis. The extracted sharp and bony foreign body was discussed among the surgical team as a sharp piece of animal bone. After patient recovery, both the patient and caretaker were interviewed in detail about the food history before the pain started. The history revealed the ingestion of home cooked buffalo meat with alcohol in the evening by the patient. The patient usually consumed buffalo meat with alcohol in the evenings. The object was confirmed as a ‘buffalo bone’ by both the patient and the caretaker (Figs. 1, 2).

## Discussion

The diagnosis of the patient based on the history, clinical examination and laboratory investigation initially pointed towards acute appendicitis. The symptoms were not typical only of acute appendicitis; however, due to lack of enough evidence, other differential diagnosis could not be considered immediately. Retrospectively, the pain can be discussed to be caused primarily by the progression of the perforation. The released intestinal contents may have collected in the right iliac fossa. This could have inflamed the appendix causing the symptoms that mimic appendicitis. A variety of foreign bodies such as dentures, toothpicks, chicken or fish bone and cocktail sticks have been implicated in the pathogenesis of gastrointestinal perforation [8]. Perforations are common in elderly individuals (who wear dentures), alcoholics, children and mentally challenged individuals. Less than 1 % of ingested foreign bodies perforate the bowel, and the greatest risk is with large, sharp or pointed objects. The foreign bodies usually pass through the esophagus without any complications, there is an increased risk for bowel perforations, bowel obstruction and other complications of complications [9]. Most common site for intestinal perforation is terminal ileum and rectosigmoid region [10]. In our patient perforation was at jejunum. The patient did not provide a history of ingestion of a foreign body, his admission to the hospital was due to a severe lower abdominal pain and we did not observe subdiaphragatic free air on a radiological evaluation. Patients often do not recall ingesting foreign body. The foreign body is found either during investigations or during surgery. Patients may present with abdominal pain, nausea, vomiting, fever, haematochezia or melena. Foreign-body perforation of the bowel presents as acute abdominal emergency mimicking acute appendicitis, diverticulitis or perforated peptic ulcer [11]. Free air is seen in 15.9 % of gastrointestinal perforations in the subdiaphragmatic region [10]. The foreign material perforates the intestinal wall and an inflammation occurs in this region with a granulation tissue covering intestinal wall and omentum with fibrin material. This can explain the low rate of subdiaphragmatic free air in a direct radiography [12]. Hard triangular plastic material was recovered during exploration laparotomy in jejunum perforation [13]. In a case of 3-months history of abdominal pain, fever and leucocytosis, an explorative laparotomy revealed fish bone. The case had jejunal perforation due to the fish bone [14]. A fish bone perforation mimicking acute appendicitis is also reported [15]. While fish bones are sharp and perforations are reported [14, 15], the case presented here had two small jejunal perforations due to a buffalo bone. He presented with symptoms of acute appendicitis. Perforations were repaired primarily (Additional file 1).

## Conclusions

Intestinal perforation by ingested foreign bodies should be suspected in acute abdomen. A detailed food history may be required for cases presenting with symptoms of acute appendicitis that do not have a typical pain history of appendicitis. It requires a high degree of suspicion and awareness on the part of the clinician.

## Additional file

10.1186/s13104-016-2127-y Care case report timeline.

## Abbreviations

IFB: ingested foreign bodies
ER: emergency room
