# Supplementary material for: Jejunal perforation due to ingested buffalo bone mimicking acute appendicitis
Source: BMC Res Notes. 2016 Jun 24;9:321. doi: 10.1186/s13104-016-2127-y (PMC4919848; doi:10.1186/s13104-016-2127-y)
Supplement: Supplementary file 1 — 10.1186/s13104-016-2127-y Care case report timeline. [file 13104_2016_2127_MOESM1_ESM.docx]

Day 0 = 15 December 2015

History: Patient Side

Day +3

Day +2

Patient discharged

Abdominal Drain Removed

Appendectomy was done.

Inflamed bowel with flakes and purulent collection made us explore the entire bowel.

Two small jejunal perforations around 2 mm size in the antimesenteric border approximately 250 cm proximal to ileocaecal junction was found.

**On Examination**:

Localised Tenderness and Guarding in Right Illiac Fossa

**On Investigation**:

Complete Blood Count, Urine Routine, Serum Urea, Creatinine, Sodium, Potassium,

Artetial Blood Gas, ECG, Chest X Ray: All within normal range

**Abdominal/Pelvic Ultrasonography:**

Appendix was not visualized, minimal fluid collection in right iliac fossa

**Diagnosis** of acute appendicitis was made

Day 0

Presented with Right Illiac fossa pain

Day 0

+8 hrs

Day -3

History: Clinician

epigastric pain, gradually getting worse

History of Alcohol intake with Buffalo meat ingestion

Day -4
